# Fluorescence energy transfer in quantum dot/azo dye complexes in polymer track membranes

**DOI:** 10.1186/1556-276X-8-452

**Published:** 2013-10-31

**Authors:** Yulia A Gromova, Anna O Orlova, Vladimir G Maslov, Anatoly V Fedorov, Alexander V Baranov

**Affiliations:** 1 Saint Petersburg National Research University of Information Technologies, Mechanics and Optics, Kronverkskiy pr, Saint Petersburg 197101, Russia

**Keywords:** Quantum dots, Azo dyes, FRET, Track membrane

## Abstract

Fluorescence resonance energy transfer in complexes of semiconductor CdSe/ZnS quantum dots with molecules of heterocyclic azo dyes, 1-(2-pyridylazo)-2-naphthol and 4-(2-pyridylazo) resorcinol, formed at high quantum dot concentration in the polymer pore track membranes were studied by steady-state and transient PL spectroscopy. The effect of interaction between the complexes and free quantum dots on the efficiency of the fluorescence energy transfer and quantum dot luminescence quenching was found and discussed.

## Background

For two decades, photophysical properties of complexes of semiconductor nanocrystals, or quantum dots (QDs), with organic molecules (OM) are of great interest. Creation of different structures based on quantum dots and organic molecules allows expanding significantly the area of quantum dot applications. These QD complexes are employed in sensors, catalysis, electronic devices, biology and medical studies [1-4].

In QD/OM complexes, quantum dots are commonly used as energy donors [2-5]. QDs have high extinction coefficient in a broad spectral range and a high luminescence quantum yield. Moreover, their optical transition wavelengths depend on the nanocrystal size. Therefore, it is very easy to match the QD luminescence and absorption spectrum of an appropriate energy acceptor needed for efficient resonance energy transfer. Additionally, QDs have high photostability and chemical resistance compared with organic molecules [6].

A lot of studies elucidate formation of complexes of quantum dots and organic molecules [7,8] that demonstrate effective QD/molecule energy transfer. The fluorescence resonance energy transfer (FRET) is common for such systems. As a rule, FRET in individual complexes was under consideration, but effects of interaction between the complexes and free donors on the efficiency of energy transfer, which may take place at a high donor concentration, were not well analyzed. The high donor (QDs) concentration can be realized, e.g. in porous matrices like the poly-(ethylene terephthalate) pore track membrane (PET TM) [9]. This matrix allows embedding the QDs in the loosened layer of the track pore walls. Another remarkable feature of the track membrane is the possibility to create the complexes by subsequent incorporation of QDs and molecules into the track membrane [10].

In the present work, FRET in complexes of semiconductor CdSe/ZnS quantum dots and molecules of heterocyclic azo dyes 1-(2-pyridylazo)-2-naphthol (PAN) and 4-(2-pyridylazo) resorcinol (PAR) formed at high concentration in the PET track membranes were studied. We demonstrate the evident effect of free QDs on the efficiency of FRET from QDs to the acceptor molecules.

## Methods

PET TM were obtained from the Flerov Laboratory of Nuclear Reactions, Joint Institute for Nuclear Research (Dubna, Russia). The membrane characteristics are as follows: pore diameter *d* is 0.5 *μ*m, thickness *l* is 12 *μ*m and pore density *n* is 2.9×10^7^ cm ^-2^.

Hydrophobic colloidal CdSe/ZnS QDs with 2.5-nm core diameter with luminescence at 530 nm were used. The membranes were immersed in the QD colloidal toluene solution with a QD concentration of 10^-6^ mol/L for 5 days. The membranes with embedded QDs were removed from the solution, rinsed thoroughly by toluene and dried before measurements. The concentration of QDs embedded in the wall layer along pores was estimated from their absorption spectrum taking into account the TM parameters with the assumption that the thickness of the layer with quantum dots does not exceed 200 nm, as was shown in [9].

PAN and PAR were purchased from Sigma Aldrich (St. Louis, MO, USA) and used without further purification. QD/azo dye complexes were produced both in solutions and in track membranes. QD/PAN complexes in the toluene solutions were prepared by adding PAN solutions with various concentrations (*C*_PAN_) to the QD solution with a concentration (*C*_QD_) of about 5×10^-7^ mol/L. The molar ratio (*n*=*C*_QD_:*C*_PAN_) in the solutions varied from 5:1 to 1:10. The QD/PAR complexes were created similarly. For creation of QD/azo dye complexes in the track membranes, the TMs with embedded QDs were immersed into toluene PAN or aqueous PAR solutions with different concentrations (10^-8^÷10^-6^ mol/L) of molecules for a week. This period is enough for the establishment of chemical equilibrium between QD/azo dye complexes and free compounds. After impregnation of azo dyes, the membranes were thoroughly rinsed in toluene and dried under the ambient condition.

A spectrofluorometer, Cary Eclipse (Varian, Palo Alto, CA, USA), and a spectrophotometer, UV-Probe 3600 (Shimadzu, Kyoto, Japan), were used for measuring the steady-state photoluminescence (PL) and absorption spectra of the samples, respectively. PL was excited using a 405-nm radiation. Time-resolved PL measurements in a backscattering geometry were performed by a MicroTime 100 laser scanning luminescent microscope (PicoQuant, Berlin, Germany), with a 100-ps time resolution. A 405-nm radiation from a 5-MHz pulsed diode laser was used for PL excitation. Spectral selection of the PL was performed using a set of interference filters with a full width at half maximum (FWHM) of 10 nm in the spectral range of 480 to 590 nm. The average PL decay time was calculated using the formula:

(1) $$\langle \tau \rangle =\sum A_{i}\tau_{i}^{2}/\sum A_{i}\tau_{i},$$

where *A*_*i*_ and *τ*_*i*_ are the amplitude and decay time of the *i*th component, respectively.

## Results and discussion

### Investigation of quantum dot interaction in the track membranes

**Figure 1:**
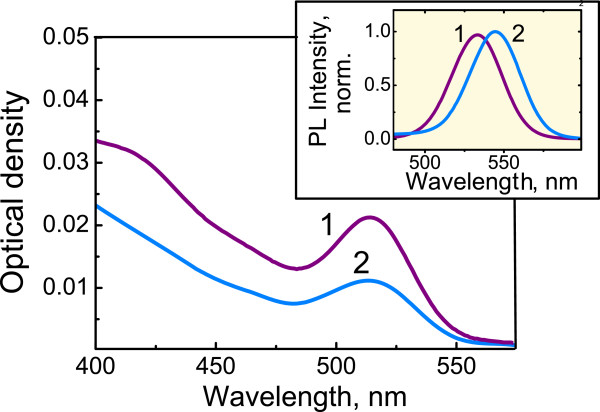
**Absorption and luminescence spectra (inset) of CdSe/ZnS quantum dots.** In toluene (1) and in track membranes (2).

**Figure 2:**
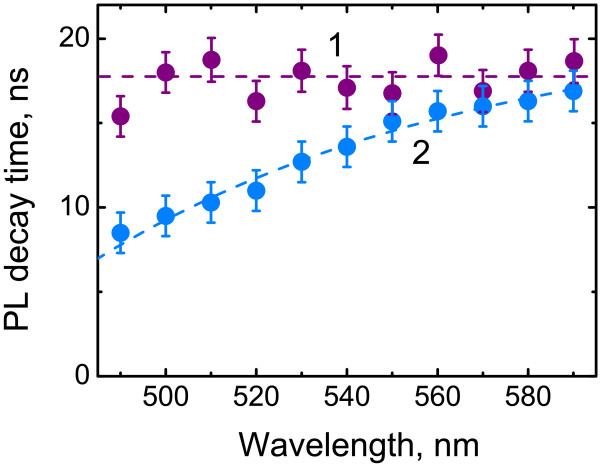
**Quantum dot photoluminescence average decay time 〈*τ*〉 as a function of detection wavelength for QDs.** In toluene solution (1) and in track membrane (2).

The absorption spectrum of QDs in the track membrane coincides with that in toluene. At the same time, a 10-nm red shift of QD PL band was observed as compared with the QDs in the toluene solution (Figure 1, inset). Local concentration of QDs in the wall layer of the TM pores of 10^-3^ mol/L estimated from the TM absorption spectrum is much higher than in the QD toluene solutions of 10^-6^ mol/L. Simple estimation, in an assumption that quantum dots are uniformly distributed within the cylindrical layer around the empty track pore, gives an average distance between QD centers of about 10 nm that makes possible an interdot FRET. Kagan et al. [11] showed that FRET between neighboring QDs in an ensemble of the nanocrystals with slightly different sizes results in red shift of the QD PL peak. Additionally, the PL decay time becomes shorter at the higher energy side of the inhomogeneously broadened QD PL band as compared with its lower energy side. We compared the decay time of the QD PL in the toluene solution (*C*_QD_ of 5×10^-7^ mol/L) and in the TM samples at different detection wavelengths using a set of narrow band interference filters. In both cases, the PL decay is well fitted by a biexponentinal function. The average luminescence decay time, 〈*τ*〉, for QDs in toluene is the same for all detection wavelengths (Figure 2, curve 1). However, in the track membranes, the PL decay time reduces three times at the higher energy side of the QD PL band as compared with the lower energy side. That indicates FRET between the QDs in the wall layer of the TM pores.

### QD/azo dye complex formation

**Figure 3:**
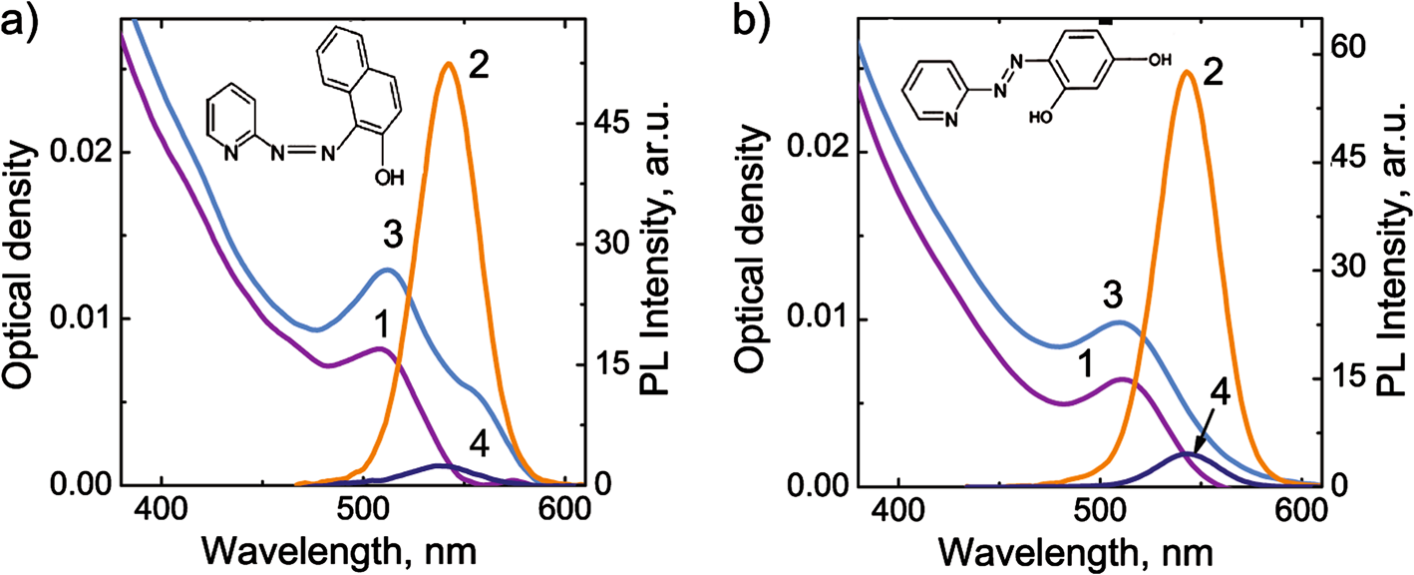
**Absorption and PL spectra of CdSe/ZnS quantum dots.** The quantum dots were embedded in track membranes before and after formation of **(a)** QD/PAN and **(b)** QD/PAR complexes. 1 and 2 are the absorbance and PL of the QD embedded in the track membrane, respectively. 3 and 4 are the absorbance and PL of those samples after their impregnation with azo dye solution, respectively.

For creation of complexes of CdSe/ZnS QDs with azo dye molecules, we employed samples of the track membrane with QDs demonstrating the interdot FRET. The azo dyes can bind with Zn ions [12], and therefore, the PAN and PAR molecules can form the QD/molecule complex by coordination onto zinc atoms on the ZnS shell of QDs. Absorption and PL spectra of track membranes with embedded CdSe/ZnS quantum dots before and after formation of QD/PAN and QD/PAR complexes are shown in Figure 3a,b, respectively. Appearance of a new absorption band at 560 nm, that is a characteristic band of PAN/Zn bonding [13], indicates the QD/PAN complex formation. Unlike PAN, the PAR is the hydrophilic molecule. Therefore, creation of its complexes with hydrophobic QDs in solution is a nontrivial task. At the same time, it was found in [10] that sequential embedding of hydrophobic CdSe/ZnS QDs and PAR molecules into track membranes leads to formation of PAR/QD complexes due to coordination of PAR molecules onto the surface Zn ions on the ZnS shell of CdSe/ZnS QDs. Figure 3b shows that a new characteristic absorption band at 520 nm in the absorption spectrum of TM treated by QDs and PAR manifests a formation of the QD/PAR complexes.

In both cases, the formation of QD/PAN and QD/PAR complexes is accompanied by QD luminescence quenching (Figure 3a,b). The QD luminescence quenching due to the efficient intracomplex FRET from QDs to these azo dye molecules has been reported in [10].

**Figure 4:**
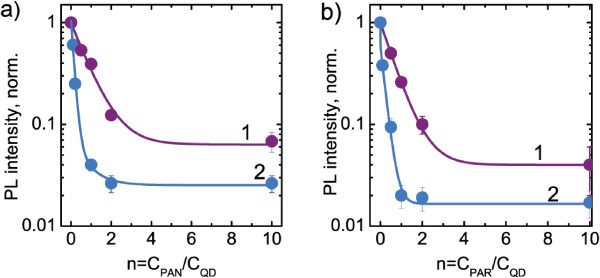
**Dependences of QD luminescence intensity on the molar ratio of azo dyes to quantum dots (**$n=C_{\text{Azo-Dye}/C_{\text{QD}}}$**).** In solutions (1) and in track membranes (2). Data for PAN and PAR azo dyes are shown in **(a)** and **(b)**, respectively.

Dependencies of the PL intensity of QDs on the molar ratio of azo dyes to quantum dots (*n*=*C*_AD_/*C*_QD_) in solutions and TMs are presented in Figure 4. It is clearly seen that the formation of QD/azo dye complexes in the TMs leads to more efficient quenching of PL of quantum dots than that in the solutions.

**Figure 5:**
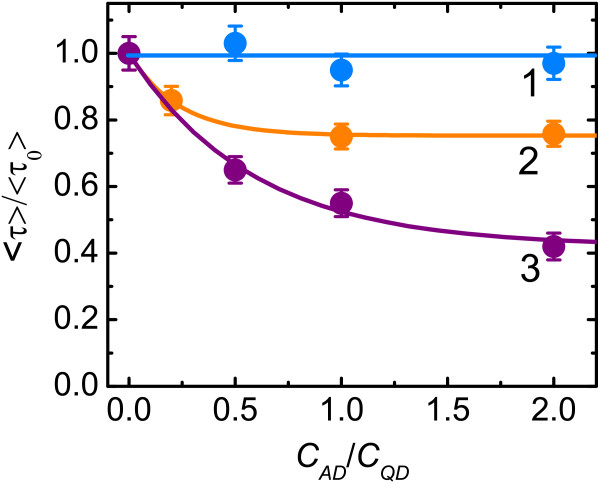
**Dependences of normalized QD average PL decay time on molar ratio of azo dyes to QDs.** In solution (1) and in track membrane for QD/PAN complexes (2) and QD/PAR complexes (3).

We observed that the QD PL decay time does not depend on the azo dye concentration (Figure 5, curve 1) in solutions and its value coincidences with the PL decay time of the free QDs. Therefore, a luminescence of unbound quantum dots is only observed in solutions, because binding at least one PAN or PAR molecule onto the QD surface leads to the total quenching of luminescence of the QD. In contrast to solutions, the increasing of azo dye concentration in the TMs was accompanied by shortening of the average QD PL decay time as shown in Figure 5. This indicates that there are some partially quenched QDs in the TMs with QD/azo dye complexes. The observed difference between QD PL decay times in the solution and TM can be explained by the fact that azo dyes bound with Zn ions on the surface of the QD quench not only the PL of this QD but also partially the luminescence of neighboring free QDs. This becomes possible because of high local concentration of QDs in the TM.

We propose a simple model of QD PL quenching in the QD/azo dye complexes. There are several assumptions in the model: 

1. The number of azo dye molecules bounded to the quantum dot surface obeys a Poisson statistic [14].

2. The probability of binding *x* azo dye molecules with the QD surface is

(2) $$\begin{array}{lcr} p(t,x) & = & e^{-t}\frac{t^{x}}{x!}, \end{array}$$

where *t* is the average number of azo dye molecules per QD.

3. The PL of QD totally quenched in the complex. Therefore, only luminescence of free QDs is observed.

4. While only free QDs luminesce, the PL intensity of QDs is proportional to the number of free QDs. Then, *x*=0 in Equation 2, and the QD PL quenching is described by

(3) $$\begin{array}{lcr} \frac{I}{I_{0}} & = & e^{-\frac{C_{\text{AD}}}{C_{\text{QD}}}}, \end{array}$$

where *I*_0_ and *I* are the PL intensity of QDs before and after QD/azo dye complex formation, respectively; *C*_AD_ and *C*_QD_ are the molar concentration of azo dye molecules and QDs, respectively.

As can be seen from Figure 4, there is a residual level of QD PL, which is independent on azo dye concentration. It indicates that not all QDs form complexes with azo dye molecules at the present experimental conditions. Let us denote the fraction of QDs involved in complex formation as *A* (0 < *A* < 1), and then 1-*A* is the fraction of free QDs. In this case, Equation 3 can be transformed as follows:

(4) $$\frac{I}{I_{0}}=Ae^{-\frac{C_{\text{AD}}}{C_{\text{QD}}}}+(1-A)=Ae^{-n}+(1-A),$$

where *n* = *C*_AD_/*C*_QD_.

Analysis of experimental data has shown that our data are well fitted by a function *f* = *A*·*f*exp(-*B*·*n*) + (1 - *A*), where *A* and *B* are the adjustable parameters. Results of fitting are listed in the Table 1. Parameter *B* contains useful information about the efficiency of PL quenching of one QD by one azo dye molecule. The *B* value close to unity indicates that the QD is totally quenched by one azo dye molecule. If the *B* value is less than unity, then the azo dye molecule quenches QD partially. If the *B* value is more than unity, this indicates that one azo dye molecule quenches more than one QD. The last case can be realized, e.g. in QD aggregates or systems with close QDs.

**Table 1:** The adjustable parameters of fitting of QD PL quenching curves presented in Figure 4

	***A***	***B***	**1-*A***
QD/PAN in solution	0.92±0.05	0.91±0.11	0.08±0.05
QD/PAN in TM	0.95±0.05	3.71±0.15	0.05±0.05
QD/PAR in solution	0.94±0.05	1.12±0.12	0.06±0.05
QD/PAR in TM	0.96±0.05	5.56±0.05	0.04±0.05

The fitting gives a *B* of about 1 for QD/azo dye complexes in solutions which means formation mainly of one azo dye molecule to one QD complex. In TMs, however, the *B* values are more than unity which indicates that one azo dye molecule totally quenches the PL of QD in the complex and at the same time partially quenches some neighboring free QDs. The *B* values of approximately 3 and approximately 5 for QD/PAN or QD/PAR complexes in track membranes show quenching of luminescence of the host quantum dot and two and four neighboring QDs, respectively.

Experimental data show that the formation of QD/PAR complexes in TMs quenches luminescence of QDs more efficiently than that of QD/PAN complexes. Simple estimations show that it is caused by different values of the corresponding overlap integral:

(5) $$\begin{array}{lcr} J & = & \int I_{D}^{H}(\nu )\cdot \varepsilon_{A}(\nu )\cdot \nu^{-4}\cdot \mathrm{d\nu}, \end{array}$$

where$I_{D}^{H}(\nu )$ is the normalized luminescence spectrum of the donor (QDs), *ε*_*A*_(*ν*) is the absorption spectrum of the acceptor (QD/azo dye complex) and *ν* is the wavenumber [14]. The overlap integral defines the critical radius (the distance between the donor and acceptor in which the FRET efficiency is 50

(6) $$\begin{array}{lcr} R_{0}^{6} & = & \frac{9000\cdot \ln10\cdot \Phi^{2}\cdot q_{0D}}{128\cdot \pi^{5}\cdot n^{4}\cdot N_{A}}\cdot J, \end{array}$$

where *Φ* is the orientation factor, *q*_0*D*_ is the quantum yield of the donor in the absence of the quencher, *n* is the refractive index of the environment and *N*_A_ is the Avogadro number.

The calculated values of overlap integrals and the critical radii of QD/azo dye donor-acceptor pairs are listed in Table 2. As clearly seen from Table 2, the quenching of QD luminescence in TMs with QD/PAR complexes is expected to be more effective than in the case of QD/PAR complexes that is in complete agreement with our experimental data.

**Table 2:** FRET parameters of QD/azo dye donor-acceptor pairs

	**Overlap integral**	**Critical radius**
	***J*, M**^**-1**^** cm**^**4**^	***R***_**0**_**, Å**
QD/PAN	5×10^-14^	31
QD/PAR	7×10^-13^	48

## Conclusion

We show that in the polymer track membranes with high local QD concentration, an efficient FRET between neighboring QDs takes place. The strong QD PL quenching is observed at additional embedding of azo dye molecules into the membranes due to formation of QD/azo dye complexes with FRET from QD to molecules. A detailed analysis of steady-state and transient QD PL responses as a function of azo dye concentration shows that one azo dye molecule bound with Zn ions on the surface of QD quenches not only PL of the QD in the complex but also PL of the closest neighboring free QDs. This becomes possible because of high local concentration of QDs in the membranes. Then, the quenching of luminescence of the QD ensemble occurs even at a relatively low complex concentration. Using different azo dyes allows to control the value of the overlap integral and to manage the efficiency of QD luminescence quenching. The present result could be used for designing different types of micro fluidic devices [13]. Dissociative sensors developing based on track membranes and QD/azo dye complexes are promising. In this system, elimination of one quencher molecule will lead to recovering luminescence signal from several QDs at once which will significantly increase sensor sensitivity.

## Competing interests

The authors declare that they have no competing interests.

## Authors’ contributions

All authors contributed equally. All authors read and approved the final manuscript.
